# Psychosocial Determinants of Work Ability Among Paramedics: Implications for Occupational Health—Pilot Study

**DOI:** 10.3390/jcm14248805

**Published:** 2025-12-12

**Authors:** Kornelia Sokołowska, Olga Fedorowicz, Janina Kulińska, Łukasz Rypicz

**Affiliations:** 1 Graduate Faculty of Health Sciences, Wroclaw Medical University, 50-367 Wrocław, Poland; sokol.kornelia2@gmail.com; 2 Department and Institute of Clinical Pharmacology, Faculty of Farmacy, Wroclaw Medical University, 50-367 Wrocław, Poland; olga.fedorowicz@umw.edu.pl; 3 Department of Public Health, Faculty of Health Sciences, Wroclaw Medical University, 50-367 Wrocław, Poland; lukasz.rypicz@umw.edu.pl

**Keywords:** paramedics, work ability, occupational stress, burnout, physical activity, psychosocial factors, emergency medical services, occupational health, workforce sustainability, public health

## Abstract

**Background/Objectives:** Paramedics are routinely exposed to high psychosocial strain due to the demanding and unpredictable nature of emergency medical work. This study aimed to examine psychosocial and behavioral factors—working hours, stress, burnout, and physical activity—associated with self-reported work ability among paramedics in Poland. **Methods:** A cross-sectional online survey was conducted between July 2023 and January 2024 among paramedics—whether active in Emergency Medical Services or holding a second degree and employed as a nurse—using the Polish version of the Work Ability Index and a stress and burnout assessment tool recommended by the European Commission. Statistical analyses, including Spearman correlation and group comparisons, were performed with a significance level of α = 0.05. **Results:** Work ability correlated positively with physical activity and negatively with age, stress, and burnout (*p* < 0.05). The strongest association was observed between stress and burnout (ρ = 0.837). Paramedics working in ambulance services reported significantly higher stress and burnout levels than hospital personnel (*p* = 0.001 and *p* = 0.002), although work ability did not differ by workplace. **Conclusions**: These findings indicate that psychosocial stress, burnout, and low physical activity substantially reduce work ability among paramedics, emphasizing the need for targeted preventive strategies—such as stress management, promotion of physical activity, and regulation of working hours—to support the health and sustainability of the emergency medical workforce.

## 1. Introduction

One of the biggest contemporary challenges for the safety and health of healthcare professionals is the exposure to psychosocial risks in the workplace [1,2]. The International Labour Organisation (ILO) defines psychosocial occupational hazards as ‘aspects of the organization and management of work, together with their social and environmental context, which have the potential to cause psychological, social or physical harm’ [3]. There is growing evidence that psychosocial risk factors in the workplace are associated with increased sickness absence, mental disorders, cardiovascular diseases, cancer, and musculoskeletal diseases. Physiological threats are characterized by changes in the nervous and endocrine systems, as well as in the circulatory, respiratory and digestive systems. In turn, mental threats can include anxiety, anger, impatience, impulsivity, depression, isolation, memory problems, reduced concentration, difficulty making decisions or distraction [4].

Emergency Medicine is a relatively young branch of medicine in Poland; however, it is one of its most dynamically growing fields [5]. Paramedics face numerous challenges that require both medical knowledge and mental toughness. Studies indicate that the main stressor among the paramedics is a high level of responsibility [6,7]. Daily contact with patients and their families, increasing aggression from patients, low levels of environmental control, a hierarchical system of professional dependency, and dissatisfaction with remuneration are becoming extremely burdensome aspects of the paramedic profession [6]. The demanding nature of work, characterized by rapid decision-making and exposure to traumatic events, places paramedics at an increased risk of experiencing burnout syndrome [8].

Research indicates that paramedics have lower levels of psychological well-being, significantly higher levels of stress disorders, and a three times higher prevalence of post-traumatic stress disorder compared to the standard population [9]. It is also confirmed that paramedics perceive the presence of selected symptoms indicative of professional burnout in themselves and confirm their pace as highly stressful [10,11].

Stress leading to professional burnout, poorer mental and physical well-being leads to reduced work capacity, which negatively affects both the paramedic and the patient. Social factors in the paramedic’s work environment are key to improving the quality of emergency services, so systemic and individual solutions should be aimed at building as much capacity in emergency services as possible. It is important to accurately identify the relationship between individual psychosocial factors and the paramedic’s work capacity. In numerous studies, ambulance personnel articulate their well-being needs across four key areas: organizational support, informal support, use of humor, and individual mechanisms to cope, such as detachment and external supports [12]. The cited article also emphasizes that the key areas affecting work ability and satisfaction for the paramedic are primarily stress levels and physical condition.

Despite growing international attention to the psychosocial well-being of healthcare workers, research focusing on Polish paramedics remains scarce. Given recent changes in licensure, educational pathways, and employment patterns, a deeper understanding of their psychosocial profile is necessary to inform national policy and practice. This study contributes to filling that void by offering both a descriptive and correlational analysis of work-related health indicators in this unique professional group.

Accurate identification and monitoring of existing psychosocial risks in the workplace is the basis for increasing the paramedic’s work ability and thus the quality of health services. However, it is equally important to implement preventive and corrective actions that address these risks proactively, rather than relying solely on their detection.

Ensuring proper working conditions in terms of psychosocial risks brings economic benefits to the employer, such as a reduction in sickness absence among paramedics or a drop in the number of mistakes and accidents, and from the employee’s perspective, it allows avoiding substance abuse, social isolation and sleep disorders [13].

The central research question of this study is as follows: How do psychosocial factors such as workplace stress, burnout, and physical activity relate to the work ability of Polish paramedics? The study addresses a significant gap in the existing literature, as there is a limited body of empirical, descriptive research focused specifically on EMS paramedics in Poland, despite their growing role in the healthcare system. The results are particularly relevant in light of recent structural and legislative changes in the field of emergency medicine, including the evolution of professional roles and dual qualifications—such as paramedics who are active in Emergency Medical Services or hold a second degree and are employed as nurses. These dual roles have not been thoroughly investigated in terms of their implications for well-being and job functioning.

## 2. Materials and Methods

A total of 121 healthcare workers participated in the study. This study included participants who were actively engaged in their professional roles as paramedics or nurses (paramedics who have a second degree and are employed as nurses) within the Polish healthcare system during the study period. The study was conducted between July 2023 and January 2024. The survey was distributed to medical personnel employed in various sectors of Poland’s national healthcare system, specifically targeting paramedics and nurses. The questionnaire was developed exclusively in electronic format and administered using Google Forms. The study utilized a convenience sampling strategy, targeting healthcare professionals who met the inclusion criteria and were accessible during the study period. No a priori sample size calculation was conducted, as the study was designed as a pilot and exploratory investigation to identify preliminary trends and relationships. Although the sample may not be statistically representative of all Polish paramedics, the demographic profile of the respondents (age, gender, workplace setting) is broadly consistent with available workforce data, supporting the comparability of the sample. Nevertheless, findings should be interpreted with caution, and generalizations should be made carefully due to the non-probabilistic nature of the sampling process.

Inclusion criteria were as follows: (1) active employment in the Polish healthcare system during the study period, (2) completion of professional education as a paramedic or as a paramedic with an additional nursing degree, and (3) voluntary consent to participate in the anonymous online survey. There were no exclusion criteria beyond not meeting the inclusion conditions.

The study used a questionnaire consisting of three elements:

1. A personal data form with questions about sociodemographic information;
2. The Work Ability Index, a tool developed by the Finnish Institute of Occupational Health (the Polish version was developed by Janusz Pokorski of Jagiellonian University with the authors’ permission [14,15]);
3. A questionnaire for assessing burnout risk and stress levels, recommended by the European Commission [16].

Participation in the study was entirely voluntary and anonymous. At the beginning of the survey, participants were given detailed information about the purpose of the study, the voluntary nature of their participation, and their right to withdraw from the survey at any time without providing a reason. No identifying data were collected.

The study was conducted in accordance with the principles outlined in the Declaration of Helsinki. Prior to data collection, an inquiry was submitted to a Bioethics Committee. The Committee responded that, under applicable Polish law, formal ethical approval was not required for anonymous, voluntary, online research without physical contact with respondents.

In the statistical analysis, significance tests were applied at a level of α = 0.05, which allowed for the determination of the relationship between the variables under study. Although multiple statistical comparisons were performed, no formal correction for multiple testing (such as the Bonferroni adjustment) was applied due to the exploratory nature of the study. Therefore, the obtained *p*-values should be interpreted with caution, acknowledging the increased risk of Type I error.

Statistical analysis was conducted by a professional statistician using the R program, version 3.6.1 (R Foundation for Statistical Computing: Vienna, Austria) [17].

## 3. Results

The study covered 121 paramedics. The participants had an average age of 31.32 years (SD = 9.65), a median age of 26 years, and ages ranging from 21 to 63 years. The study group was predominantly male (52.07%), with women accounting for the remaining 47.93%.

In terms of education, 65.29% of respondents had a bachelor’s degree, while 26.45% had a master’s degree. On average, respondents had worked in the profession for 7.70 years (SD = 9.30), with a median of three years and a range from 0.5 to 48 years.

More than half of the respondents (56.2%) were employed in emergency medical services. Regarding monthly working hours, the largest percentage (47.11%) worked between 160 and 240 h per month. Meanwhile, 14.88% worked between 240 and 320 h, and 4.13% worked over 320 h.

Regarding physical activity, 11.57% of respondents reported no activity at all, while 41.32% reported low activity levels. Only 9.92% had a high level of physical activity and just 2.48% had a very high level.

These data suggest that most of the surveyed paramedics are young people who have been working in the profession for a relatively short time. They have higher education qualifications and perform intensive work in terms of both hours and physical activity. At the same time, they have a low level of physical activity outside of work. Detailed sociodemographic data are presented in Table 1.

The majority of respondents (representing over 60% of the sample) rated their work ability as good (37.19%) or excellent (24.79%). 26.45% of participants reported a moderate level of work ability, while 11.57% indicated poor work ability.

Regarding stress level, the majority of participants (42.15%) fell into the elevated-risk category, while a nearly equal proportion (40.5%) were classified as high risk. Only 17.36% of respondents were identified as being at no risk, suggesting that this professional group experiences a significant psychological burden.

With respect to burnout, 47.11% of paramedics were classified as high risk, while 39.67% were classified as elevated risk. Just 13.22% of respondents reported no risk of burnout, highlighting serious concerns about the mental well-being of emergency medical services personnel. Detailed results for these variables are presented in Table 2.

Table 3 shows the results of Spearman’s rank correlation coefficient test for six variables: age (1), hours worked per month (2), physical activity (3), work ability (4), stress level (5) and burnout (6).

The results for three variables were analyzed depending on the respondents’ place of employment: in the Emergency Medical Services or in a hospital (Table 4). These variables were work capacity, stress level and burnout.

## 4. Discussion

To our knowledge, this is one of the first descriptive studies focused specifically on the psychosocial conditions and work ability of paramedics in Poland. Previous research has largely aggregated data from various healthcare professions, overlooking the specific context of EMS work. This study fills a critical gap by identifying job-related stressors and personal health behaviors that predict reduced work ability and heightened burnout risk in this unique professional group.

A survey of Polish healthcare workers reveals a complex picture of their professional, health and psychophysical situation. The high level of psychophysical stress that is characteristic of this profession requires a systematic approach to maintaining homeostasis and preventing burnout. This directly impacts the quality of care provided and patient safety.

The data obtained indicate that a significant proportion of employees (around 67%) regularly exceed standard working hours, with 4.13% performing duties equivalent to nearly two full-time jobs per month. While no significant correlation was found between the number of hours worked and self-assessment of work ability, a positive relationship was observed between workload, stress levels and increased risk of burnout. According to data from the World Health Organization (2022), working more than 55 h per week is associated with an increased risk of cardiovascular disease (including ischemic heart disease and stroke), mental health problems, and obesity [18]. These findings confirm the seriousness of the trends observed in the study and their potential impact on the health of healthcare workers.

In the context of current generational changes, it is worth noting the results of the research by Kiedik et al. (2023), which indicate that representatives of Generation Z—who constitute an increasing percentage of healthcare workers—are characterized by high expectations in terms of work–life balance and the need for emotional support in the workplace [18].

A review by Nagle et al. (2024) also highlights the negative consequences of chronic overwork, describing impaired concentration, an increased risk of errors and a decline in the quality of healthcare services provided, among other things [19].

An important aspect of the study was determining the relationship between stress and burnout. In the study group, over 40% of respondents were found to be at high risk of burnout and showed a high risk of stress. The strong correlation between these variables (ρ = 0.837; *p* < 0.05) confirms their mutual interaction and convergence. These results are consistent with the meta-analysis by Gómez-Urquiza et al. (2017), which identified occupational stress as one of the most important predictors of burnout, especially among medical workers performing their duties in conditions of high emotional and decision-making stress [20]. The strong association between stress and burnout confirms earlier findings; however, the observed disconnect between burnout levels and perceived work ability may represent a novel insight, highlighting a potential underestimation of mental strain among paramedics. Additionally, a systematic review by Li et al. (2024) demonstrated that high levels of burnout are associated with an increased risk of errors, reduced quality of care, and increased depressive symptoms among healthcare personnel [21]. The growing number of civil lawsuits in healthcare is an indirect reflection of employee overload, organizational pressure and insufficient systemic support—factors that directly affect the ability to work [22].

The level of physical activity among respondents is also a cause for concern—more than half of those surveyed reported low or no activity. Meanwhile, it has been shown that regular physical activity correlates positively with subjective assessments of work ability (ρ = 0.217; *p* < 0.05), suggesting a positive impact on professional functioning. This finding corroborates the results of global studies indicating that a lack of physical activity is a significant risk factor for health issues, including depression and anxiety [18]. As indicated by the results of a review conducted by Bischoff et al. (2019) [23], it is important to support medical staff in increasing their resilience to unavoidable occupational stress. Interventions related to physical activity are an effective way of reducing stress [23]. Zhang et al. (2021) [24] conducted a systematic review of randomized clinical trials and found that physical relaxation can help to reduce occupational stress in healthcare workers. They identified yoga as the most effective method [24].

An analysis of the differences between emergency medical service (EMS) workers and hospital staff reveals an interesting phenomenon. The nature of work in emergency medical services—which is highly dynamic and unpredictable, requires quick decision-making, and offers limited opportunities for consultation—can contribute to increased stress and a higher risk of burnout. In contrast, the hospital environment is characterized by greater organizational structure, the possibility of team support and access to training and supervision. Working in an interdisciplinary team makes it easier to share responsibility and obtain feedback, promoting learning and strengthening the sense of competence. Conversely, paramedics working in teams of two, often comprising inexperienced individuals, experience decision-making isolation and a lack of support, exacerbating their mental strain. Additionally, the absence of continuity of care and feedback on patients’ subsequent outcomes can lead to feelings of powerlessness and loss of agency, factors strongly associated with burnout.

Despite significant differences in stress and burnout levels, no differences were found in subjective assessments of work ability. This may indicate the existence of compensatory mechanisms or variations in how one perceives their own professional competence. Similar observations were made by Dyrbye et al. (2019), who pointed to a dissonance between perceived work ability and actual mental strain [25].

High stress levels and burnout risk, coupled with inadequate compensation and prevention mechanisms, can significantly impact the quality of medical care and patient safety. This study clearly shows that comprehensive, systemic measures are needed to improve paramedics’ working conditions, reduce psychosocial stress and develop preventive strategies to maintain work ability and professional well-being.

It is worth noting that, while the study identified statistically significant associations between selected variables, no multivariable statistical models were applied. This decision was intentional, reflecting the exploratory and pilot nature of the research. The goal was to detect initial patterns and relationships that could inform more complex analyses in future, larger-scale studies. Nonetheless, the multifactorial nature of burnout, stress, and work ability is well recognized, and future research should incorporate advanced modeling techniques to account for potential confounders and interaction effects.

## 5. Conclusions

Psychosocial factors, such as stress, burnout, and physical activity, have a significant influence on the work ability of paramedics. Emergency medical service personnel experience higher levels of stress and burnout than hospital staff, indicating the need for interventions to improve their working conditions. Low physical activity and multiple job commitments can exacerbate negative health outcomes and reduce professional well-being. Therefore, systemic measures should be implemented to support the mental health and resilience of healthcare workers through stress reduction, promotion of physical activity, and regulation of workload. These recommendations are directly linked to the core variables analyzed in this study—stress, burnout, physical activity, and work ability—and should be tailored to the specific realities of EMS professionals.

The results of this study highlight the necessity of practical actions to enhance paramedics’ work capacity and well-being. Recommended strategies include training in stress management to reduce burnout risk, providing access to psychological support in the workplace, introducing programs that promote regular physical activity, and monitoring working hours to prevent excessive workload and its associated health consequences. Immediate preventive actions are essential to improve safety and reduce the prevalence of stress and burnout among healthcare professionals. In particular, interventions must be specifically adapted to the high-intensity nature of emergency work, where rapid decision-making and recurrent exposure to trauma are significant psychological burdens. Addressing these job-specific stressors through structured support and training is critical to sustaining paramedics’ health and professional functioning.

## 6. Study Limitations

One limitation of the present study is the absence of formal correction for multiple statistical comparisons. Given the exploratory nature of the research, this approach was intended to allow for a broader examination of potential associations. However, this may have increased the likelihood of Type I errors, and thus, the results should be interpreted with appropriate caution.

A potential limitation is the inclusion of participants with dual qualifications (paramedic and nurse) working in hospital settings. While these individuals were trained as paramedics, their mixed roles may have influenced responses. This reflects the specific context of the Polish healthcare system, where paramedics often pursue nursing degrees due to limited postgraduate education (master’s degree) opportunities in their field. Finally, as the study was based on self-reported data, it may be subject to response bias. Future studies using longitudinal or mixed-method designs could provide a more robust assessment of causal mechanisms.

Moreover, the study utilized a convenience sampling approach. The online questionnaire was distributed informally via professional mailing lists, paramedic-focused social media groups, and within workplace settings. Due to the decentralized and voluntary nature of this distribution, it was not possible to determine how many individuals received the survey, and thus a response rate could not be calculated. This limits the ability to assess potential non-response bias. According to official statistics from the Central Statistical Office of Poland, approximately 20,255 individuals held paramedic certification in 2024, though the actual accessible population for this survey remains unknown.

## Figures and Tables

**Table 1:** Demographic and occupational characteristics of the study group.

Variable		Total (N = 121)
Age (years)	Mean (SD)	31.32 (9.65)
	Median (quartiles)	26 (24–39)
	Range	(21–63)
Sex	Female	58 (47.93%)
	Male	63 (52.07%)
Marital status	Single	30 (24.79%)
	In a relationship	86 (71.07%)
	Separated	3 (2.48%)
	Divorced	2 (1.65%)
Education	Secondary	10 (8.26%)
	Bachelor’s degree	79 (65.29%)
	Master’s degree	32 (26.45%)
Years of professional experience	Mean (SD)	7.7 (9.30)
	Median (quartiles)	3 (1.5–10)
	Range	(0.5–48)
Current workplace	Emergency Medical Service	68 (56.2%)
	University Hospital	16 (13.22%)
	Military Hospital	3 (2.48%)
	Regional Multispecialty Hospital	13 (10.74%)
	District Hospital	6 (4.96%)
	Other	15 (12.4%)
Employment type	Employment contract	68 (56.2%)
	Contract for services (B2B)	24 (19.83%)
	Contract of mandate	25 (20.66%)
	Other	4 (3.31%)
Position *	Paramedic	88 (72.73%)
	Nurse	27 (22.31%)
	Other	6 (4.96%)
Number of hours worked per month	Up to 80 h	4 (3.31%)
	80 to 160 h	37 (30.58%)
	160 to 240 h	57 (47.11%)
	240 to 320 h	18 (14.88%)
	Over 320 h	5 (4.13%)
Physical activity	No physical activity	14 (11.57%)
	Low level of activity	50 (41.32%)
	Moderate level of activity	42 (34.71%)
	High level of activity	12 (9.92%)
	Very high level of activity	3 (2.48%)
Do you work in more than one place?	Yes	62 (51.24%)
	No	58 (47.93%)
	I am currently working outside my profession	1 (0.83%)

* Until now, the Polish medical education system only offered bachelor’s degree programs in emergency medical services, not master’s degree programs. Some people who have completed medical rescue studies have also completed nursing studies, meaning they have two medical qualifications. This is why nurses appear alongside paramedics in the table. In October 2025, it will be possible to pursue master’s degree studies in medical rescue.

**Table 2:** Distribution of participants according to work ability, stress level, and occupational burnout.

Variable		Total (N = 121)
Work ability	Poor	14 (11.57%)
	Moderate	32 (26.45%)
	Good	45 (37.19%)
	Excellent	30 (24.79%)
Stress level	No risk	21 (17.36%)
	Elevated risk	51 (42.15%)
	High risk	49 (40.5%)
Burnout	No risk	16 (13.22%)
	Elevated risk	48 (39.67%)
	High risk	57 (47.11%)

**Table 3:** Spearman’s rank correlation matrix between the studied variables.

Variable	(1)	(2)	(3)	(4)	(5)	(6)
Age (1)	-	0.001	−0.135	−0.348 *	0.207 *	0.123
Number of hours worked per month (2)		-	−0.098	0.148	0.206 *	0.145
Physical activity (3)			-	0.217 *	−0.010	0.104
Work ability (4)				-	−0.218 *	−0.185 *
Stress level (5)					-	0.837 *
Burnout (6)						-

Spearman’s correlation coefficient. * Statistically significant result (*p* < 0.05).

**Table 4:** A Comparison of work capacity, stress levels and burnout, depending on the place of employment, is presented.

Variable	Current Workplace	N	Mean	SD	Median	Min	Max	Q1	Q3	*p*
Work ability	Emergency Medical Services	68	37.75	6.88	39	24	48	33	43	*p* = 0.872
	Hospital	38	37.84	7.39	39	21	49	32.5	45	
Stress	Emergency Medical Services	68	10.09	3.14	10.5	4	15	8	13	*p* = 0.001 *
	Hospital	38	7.39	3.94	7.5	0	15	4.75	10.25	
Burnout	Emergency Medical Services	68	10.74	3.37	12	0	15	8.25	13	*p* = 0.002 *
	Hospital	38	8.13	4.35	9	0	15	6.5	12	

Mann–Whitney test; SD: standard deviation; Q1: lower quartile; Q3: upper quartile. * Statistically significant result (*p* < 0.05).

## Data Availability

The raw data supporting the conclusions of this article will be made available by the authors on request.
